# The Retinoic Acid-Metabolizing Enzyme Cyp26b1 Regulates CD4 T Cell Differentiation and Function

**DOI:** 10.1371/journal.pone.0072308

**Published:** 2013-08-22

**Authors:** Alistair Chenery, Kyle Burrows, Frann Antignano, T. Michael Underhill, Martin Petkovich, Colby Zaph

**Affiliations:** 1 The Biomedical Research Centre, University of British Columbia, Vancouver, British Columbia, Canada; 2 Department of Pathology and Laboratory Medicine, University of British Columbia, Vancouver, British Columbia, Canada; 3 Department of Cellular and Physiological Sciences, University of British Columbia, Vancouver, British Columbia, Canada; 4 Department of Biomolecular and Medical Sciences, Cancer Research Institute, Queen’s University, Kingston, Ontario, Canada; McGill University, Canada

## Abstract

The vitamin A metabolite retinoic acid (RA) has potent immunomodulatory properties that affect T cell differentiation, migration and function. However, the precise role of RA metabolism in T cells remains unclear. Catabolism of RA is mediated by the Cyp26 family of cytochrome P450 oxidases. We examined the role of Cyp26b1, the T cell-specific family member, in CD4^+^ T cells. Mice with a conditional knockout of Cyp26b1 in T cells (*Cyp26b1* ^−/−^ mice) displayed normal lymphoid development but showed an increased sensitivity to serum retinoids, which led to increased differentiation under both inducible regulatory T (iT_reg_) cell- and T_H_17 cell-polarizing conditions *in vitro*. Further, *Cyp26b1* expression was differentially regulated in iT_reg_ and T_H_17 cells. Transfer of naïve *Cyp26b1* ^−/−^ CD4^+^ T cells into *Rag1* ^−/−^ mice resulted in significantly reduced disease in a model of T cell-dependent colitis. Our results show that T cell-specific expression of Cyp26b1 is required for the development of T cell-mediated colitis and may be applicable to the development of therapeutics that target Cyp26b1 for the treatment of inflammatory bowel disease.

## Introduction

Retinoic acid (RA) is a vitamin A metabolite that plays a critical role during embryonic development [1] and has important immunomodulatory functions in adults [2]. For example, vitamin A deficiency can lead to profound immunological impairments in children such as an increased susceptibility to infections [3]. RA binds nuclear RA receptors (RARs) and retinoid X receptors which transcriptionally regulate genes that contain specific RA response elements [4]. Synthesis of RA from vitamin A is a tightly controlled process that enables specialized cells such as dendritic cells (DCs) to modulate the activation, gut homing ability and function of CD4^+^ T cells [5]. Additionally, RA has been shown to strongly promote the differentiation of inducible regulatory T cells (iT_reg_ cells) in the presence of TGF-β [6] and can modulate the migration and function of T helper 17 (T_H_17) cells in the intestine [7]. Interestingly, it has recently been shown that RA signaling occurs in T cells during the early stages of inflammation [8], suggesting that RA may be required for optimal effector T cell responses. Indeed, optimal T_H_1 effector T cell responses during *Toxoplasma gondii* infection require RA signaling [9]. Thus, RA signaling is critical for both effector and regulatory T cell function.

Despite the importance of RA signaling in T cells, very little is known about the molecular mechanisms that control RA bioavailability, signaling and metabolism in T cells and how these processes ultimately affect T cell differentiation and function. The cytochrome P450 family 26, subfamily b, polypeptide 1 (Cyp26b1) enzyme has been recently identified as the primary negative regulator of RA responsiveness in T cells [10]. Cyp26b1 is highly induced in the presence of RA and is downregulated by the cytokine TGF-β1 [10]. Cyp26b1 was also shown to modulate the RA-dependent expression of the gut-homing receptor CCR9 on T cells [10]. Thus, regulation of RA signaling by Cyp26b1 likely plays a central role in T cell function. However, the specific role of Cyp26b1 in T cells has not been investigated *in vivo*.

RA has been shown to play an important role in mucosal immune responses and oral tolerance. Intestinal cell populations including DCs and epithelial cells are significant sources of RA [2] and vitamin A deficiency has a dramatic effect on intestinal immunity and physiology [11]. Further, administration of RA can protect mice from chemically-induced colitis and the ratio of Foxp3 to IL-17 expression increased in colon biopsies treated with RA from ulcerative colitis patients [12]. The aim of the present study was to assess the role of Cyp26b1 in regulating RA-dependent T cell immune responses in the intestine. We show that although T cell-intrinsic expression of Cyp26b1 is dispensable for T cell development, Cyp26b1-deficient T cells display enhanced iT_reg_ and T_H_17 cell differentiation *in vitro*. Further, following adoptive transfer into immunodeficient hosts, Cyp26b1-deficient T cells induced significantly less intestinal inflammation. Together, our results identify a critical role for Cyp26b1-dependent catabolism of RA in T cell differentiation and function.

## Results

### Expression of Cyp26b1 is Dispensable for T Cell Development

**Figure 1:**
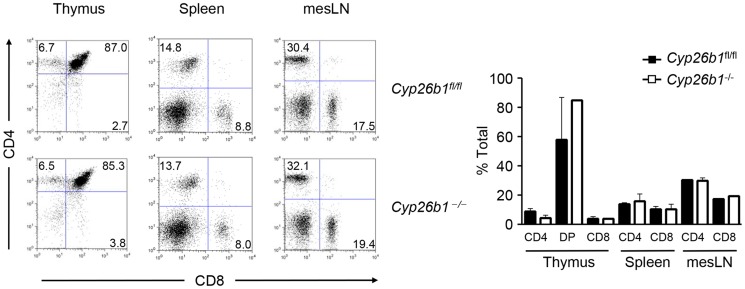
Cyp26b1 is dispensable for normal lymphoid development. *Cyp26b1* was specifically deleted in T cells. Thymus, spleen and mesenteric lymph nodes (mesLNs) from *Cyp26b1* ^fl/fl^ and *Cyp26b1* ^−/−^ mice were analyzed for CD4^+^ and CD8^+^ cell frequencies by flow cytometry. Data are from one representative experiment of 2 independent experiments (n = 3–4 per experiment).

Mice with a germline deletion of Cyp26b1 display severe bone and limb abnormalities and die *in utero* [14]. In order to assess the role of Cyp26b1 in adult T cells, we generated mice with a T cell-specific deletion of *Cyp26b1* by breeding *Cyp26b1* ^fl/fl^ mice with mice expressing the *Cre* recombinase under the control of the *Cd4* promoter/enhancer (here termed *Cyp26b1* ^−/−^ mice). *Cyp26b1* ^−/−^ mice developed normally into adult-hood, displayed no gross defects and were born with expected Mendelian ratios compared to *Cyp26b1* ^fl/fl^ littermates. We failed to observe any differences in the frequency of CD4^+^ and CD8^+^ single-positive, or CD4^+^CD8^+^ double-positive thymocytes in *Cyp26b1* ^−/−^ mice **(** **Figure 1** **)**. Further, *Cyp26b1* ^−/−^ mice had equivalent frequencies of CD4^+^ and CD8^+^ cells in the spleen and mesenteric lymph nodes (mesLN) compared to *Cyp26b1* ^fl/fl^ mice. Thus, Cyp26b1 is not required for naïve T cell development in the thymus or periphery.

### Naturally-occurring T_reg_ Cells are Normal in *Cyp26b1^−/−^* Mice

**Figure 2:**
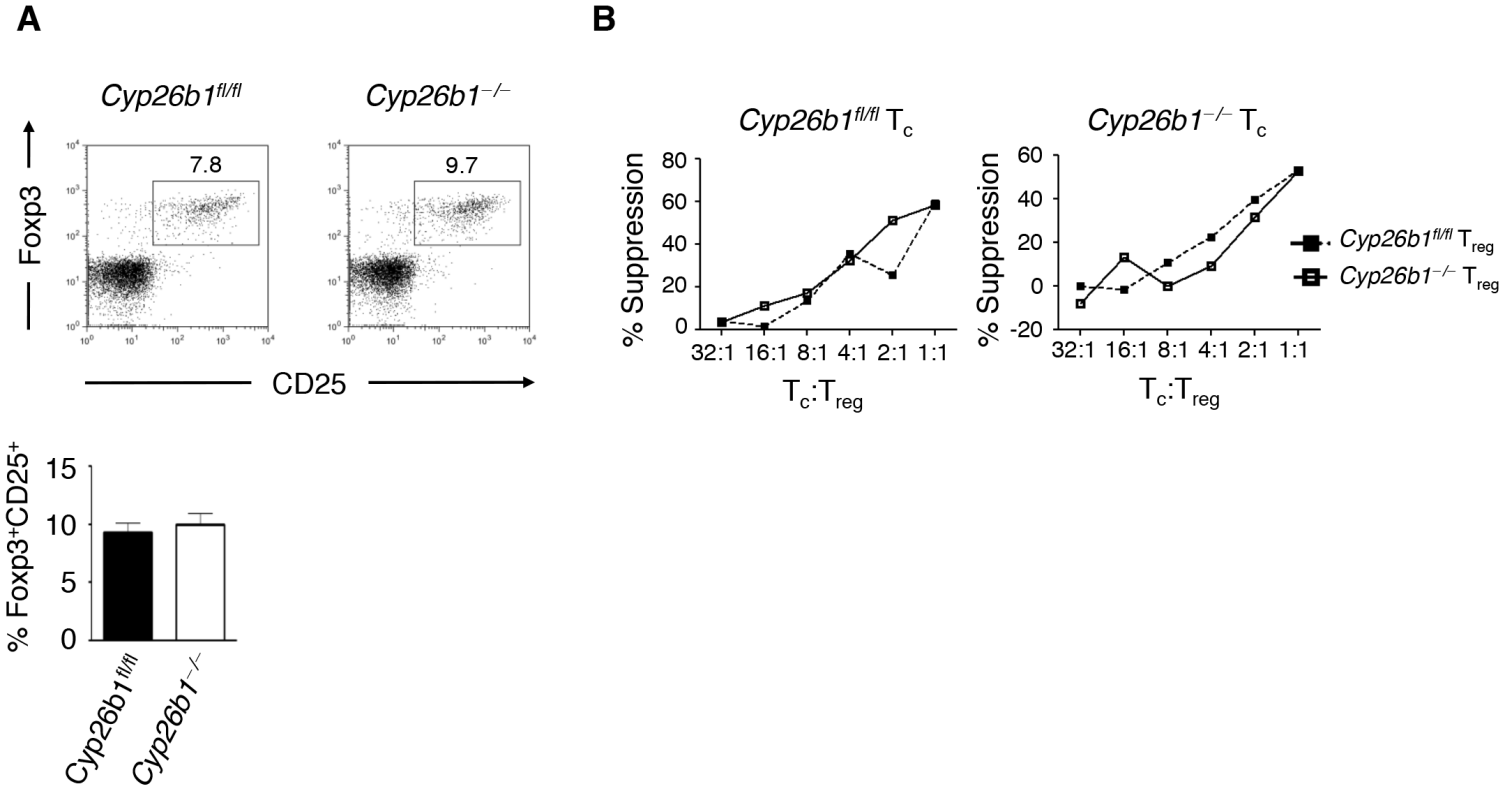
Cyp26b1 is not required for nT_reg_ cell development and suppressive function. T cells were isolated from spleens of *Cyp26b1* ^fl/fl^ and *Cyp26b1* ^−/−^ mice. (**A**) CD4^+^Foxp3^+^CD25^+^ nT_reg_ cell frequencies were determined by flow cytometry. (**B**) Purified CD4^+^CD25^+^ nT_reg_ were co-cultured with CFSE-labeled CD4^+^CD25^−^ conventional T (T_c_) cells at increasing ratios and suppression of T_c_ cells was measured by flow cytometry. Data in (**A**) are from one representative experiment of 3 independent experiments (n = 3−4 per experiment); Data in (**B**) are from a single experiment.

The role of RA signaling in the development of thymic-derived naturally-occurring T_reg_ (nT_reg_) cell development has not been examined in detail, although RAR-activating retinoids have been shown to be produced within the thymus [15]. We examined the frequency and function of nT_reg_ cells in *Cyp26b1* ^−/−^ mice. We observed equivalent frequencies of nT_reg_ cells in the spleens of *Cyp26b1* ^fl/fl^ and *Cyp26b1* ^−/−^ mice **(** **Figure 2A** **)**, suggesting that RA signaling is not a major determinant of nT_reg_ cell development. Further, Cyp26b1 was dispensable for the suppressive ability of nT_reg_ cells **(** **Figure 2B** **)**, as nT_reg_ cells from either *Cyp26b1* ^fl/fl^ or *Cyp26b1* ^−/−^ mice were able to suppress effector T cell proliferation equivalently. Thus, Cyp26b1-dependent RA metabolism is not required for nT_reg_ cell development and suppressive function.

### Cyp26b1 Modulates iTreg and TH17 Cell Polarization *In Vitro*

**Figure 3:**
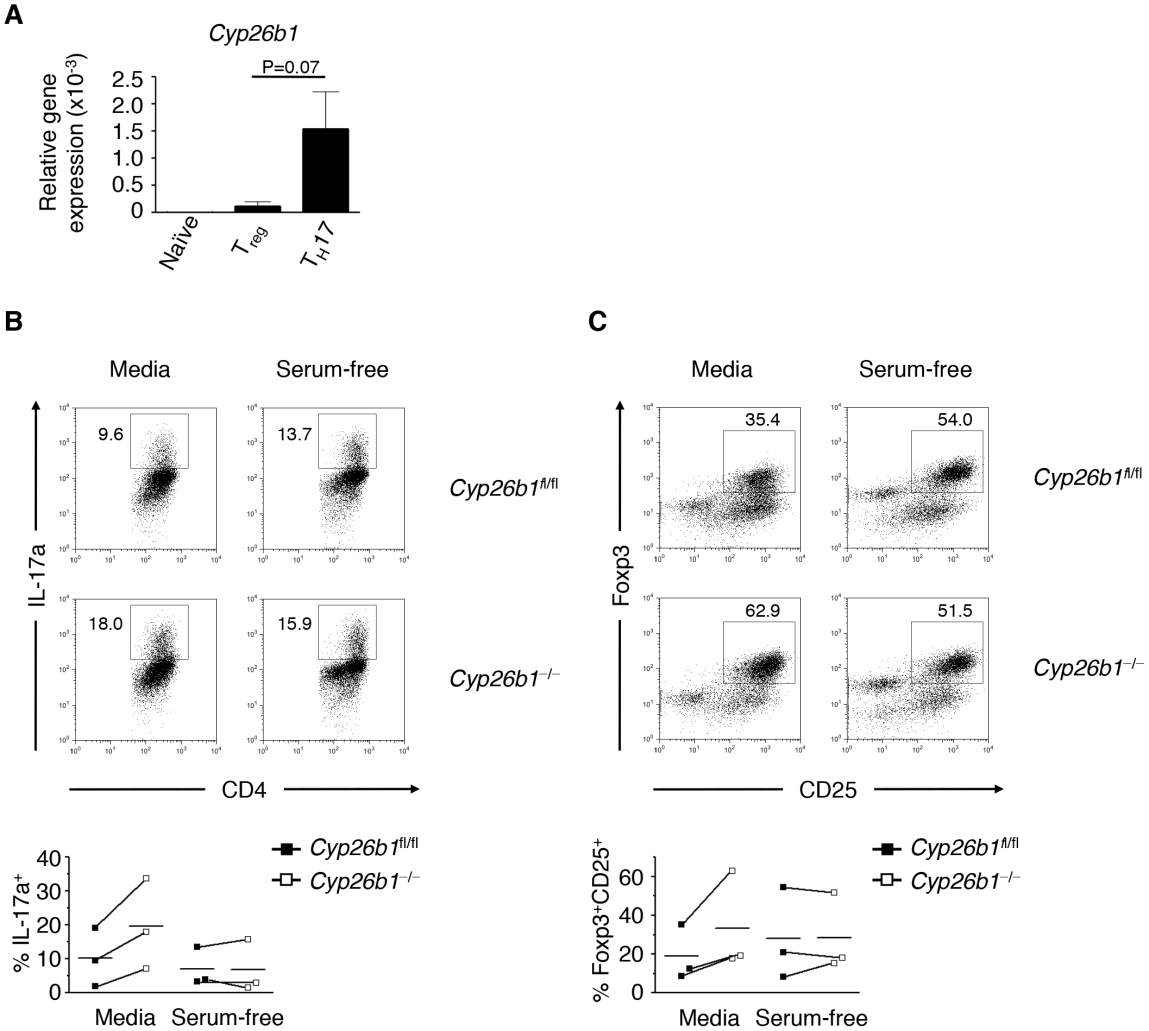
Cyp26b1 limits iT_reg_ and T_H_17 cell differentiation *in vitro*. CD4^+^ T cells were isolated from *Cyp26b1* ^fl/fl^ and *Cyp26b1* ^−/−^ mice and cultured in iT_reg_ cell- or T_H_17 cell-promoting conditions, in either serum-containing media or in serum-free media. (**A**) Gene expression of *Cyp26b1* (normalized relative to *Actb*) was measured by qRT-PCR. (**B**) Frequencies of IL-17a^+^ T_H_17 cells and (**C**) Foxp3^+^ CD25^+^ iTreg cells were determined by flow cytometry. Data in (**A**) represent mean±SEM of 4 independent experiments; Data in (**B**) and (**C**) are from one representative experiment of 4 independent experiments.

RA plays an important role in the differentiation of naive CD4^+^ T cells into iT_reg_ and T_H_17 cells [6], [7], [16]. To directly test whether Cyp26b1-dependent regulation of RA signaling controls iT_reg_ or T_H_17 cell differentiation, we stimulated CD4^+^ T cells from *Cyp26b1* ^fl/fl^ and *Cyp26b1* ^−/−^ mice under iT_reg_ cell- and T_H_17 cell-promoting conditions. Increased expression of *Cyp26b1* was observed in T_H_17 cells, with a lower expression in iT_reg_ cells **(** **Figure 3A** **)**. Following stimulation under T_H_17 cell-promoting conditions, we observed a marked increased frequency of IL-17a-producing CD4^+^ T cells in the absence of Cyp26b1 **(** **Figure 3B** **)**. These results are consistent with the expression pattern of Cyp26b1 and suggest that induction of Cyp26b1 is required for limiting T_H_17 cell differentiation. Surprisingly, we also found that the absence of Cyp26b1 resulted in heightened frequencies of CD4^+^CD25^+^Foxp3^+^ iT_reg_ cells **(** **Figure 3C** **)**, despite the low levels of *Cyp26b1* expression observed in iT_reg_ cells. These results suggest that metabolism of RA is important for limiting iT_reg_ and T_H_17 cell responses. However, we had not added any exogenous RA to these cultures, suggesting that low levels of serum retinoids affect iT_reg_ and T_H_17 cell differentiation in the absence of Cyp26b1. To directly test this, we repeated the experiment in serum-free media. Under these conditions, we found equivalent frequencies of iT_reg_ cells and T_H_17 cells following stimulation of CD4^+^ T cells from both *Cyp26b1* ^fl/fl^ and *Cyp26b1* ^−/−^ mice. Thus, Cyp26b1 regulates RA signaling in T cells and is critical for limiting iT_reg_ and T_H_17 cell differentiation. Further, these results suggest that physiological levels of RA in naïve cells impacts effector T cell differentiation.

### Cyp26b1-deficient T Cells Fail to Induce Intestinal Inflammation

**Figure 4:**
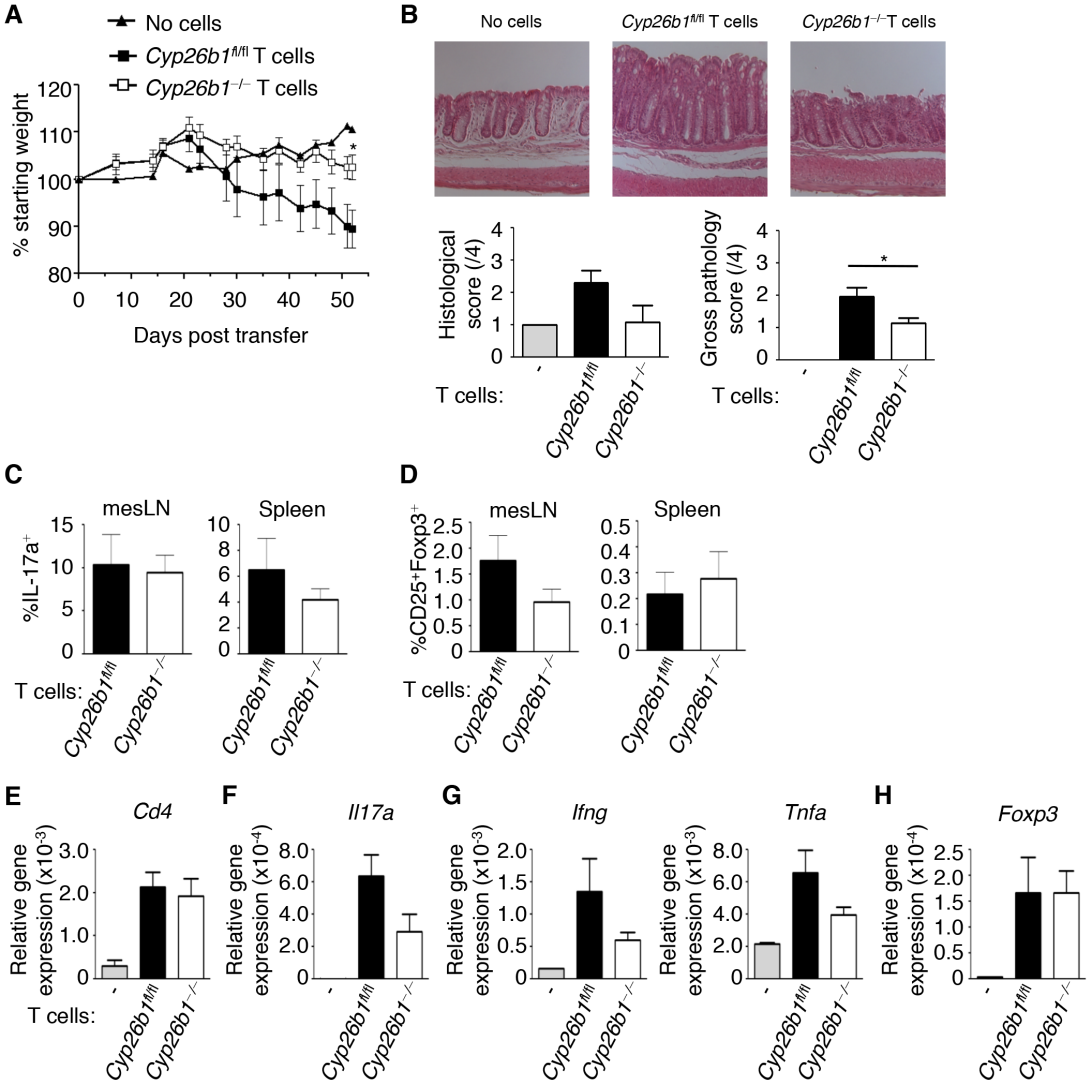
Cyp26b1-deficient T cells fail to promote intestinal inflammation following adoptive transfer into *Rag1^−/−^* mice. CD4^+^CD45RB^high^CD25^−^ naïve effector T cells from *Cyp26b1* ^fl/fl^ or *Cyp26b1* ^−/−^ mice were transferred i.p. into *Rag1* ^−/−^ mice. (**A**) Weight loss was monitored over the entire disease course. (**B**) Histological sections of proximal colons stained with hematoxylin and eosin were scored for pathology and colons were scored for gross pathology. Cells from spleens and mesLNs were polyclonally stimulated overnight and stained for (**C**) IL-17a and (**D**) Foxp3 and CD25, then measured by flow cytometry. Gene expression of (**E**) *Cd4*, (**F**) *Il17a*, (**G**) *Ifng* and *Tnfa*, and (**H**) *Foxp3* (normalized relative to *Actb*) in proximal colons was measured by qRT-PCR. Data in (**A–H**) are representative of one of 2 independent experiments (n = 7−9 per experiment).

Based on our *in vitro* results demonstrating a role for Cyp26b1 in limiting iT_reg_ and T_H_17 cell differentiation, we next examined the role of Cyp26b1 in T cell differentiation *in vivo.* We employed a well-characterized model of T cell-dependent intestinal inflammation [17]. Transfer of CD4^+^CD45RB^high^CD25*^−^* naïve T cells isolated from *Cyp26b1* ^fl/fl^ mice into immunodeficient *Rag1* ^−/−^ mice resulted in significant weight loss and morbidity associated with intestinal inflammation **(** **Figure 4A,B** **)**. In contrast, transfer of T cells isolated from *Cyp26b1* ^−/−^ mice resulted in significantly attenuated disease progression, including decreased weight loss and less severe intestinal inflammation. In contrast to our *in vitro* results, polyclonal stimulation of cells isolated from mesLNs or spleens resulted in no striking differences in the production of IL-17a by *Cyp26b1* ^fl/fl^ and *Cyp26b1* ^−/−^ T cells **(** **Figure 4C** **)**. In addition, the frequency of Foxp3^+^ T_reg_ cells in the mesLN and spleen of *Rag1^−/−^*mice that received either *Cyp26b1* ^fl/fl^ or *Cyp26b1* ^−/−^ T cells were also similar **(** **Figure 4D** **)**. Despite equivalent numbers of *Cyp26b1* ^fl/fl^ and *Cyp26b1* ^−/−^ CD4^+^ T cells in the intestine as measured by *Cd4* gene expression **(** **Figure 4E** **)**, we observed reduced levels of *Il17a* gene expression in *Rag1* ^−/−^ mice that received *Cyp26b1^−/−^* T cells **(** **Figure 4F** **)**. Further, consistent with reduced disease, we observed decreased expression of the pro-inflammatory cytokines *Ifng* and *Tnfa* in the intestine of *Rag1* ^−/−^ mice that received *Cyp26b1* ^−/−^ T cells **(** **Figure 4G** **)**. The reduced capacity of *Cyp26b1* ^−/−^ T cells to cause disease was not due to an increase in T_reg_ cells in the intestine, based on *Foxp3* gene expression **(** **Figure 4H** **)**. Thus, Cyp26b1 is critical for the development of pathological T cell responses in the intestine.

#### Cyp26b1-deficient T cells do not have altered intestinal homing molecule expression

**Figure 5:**
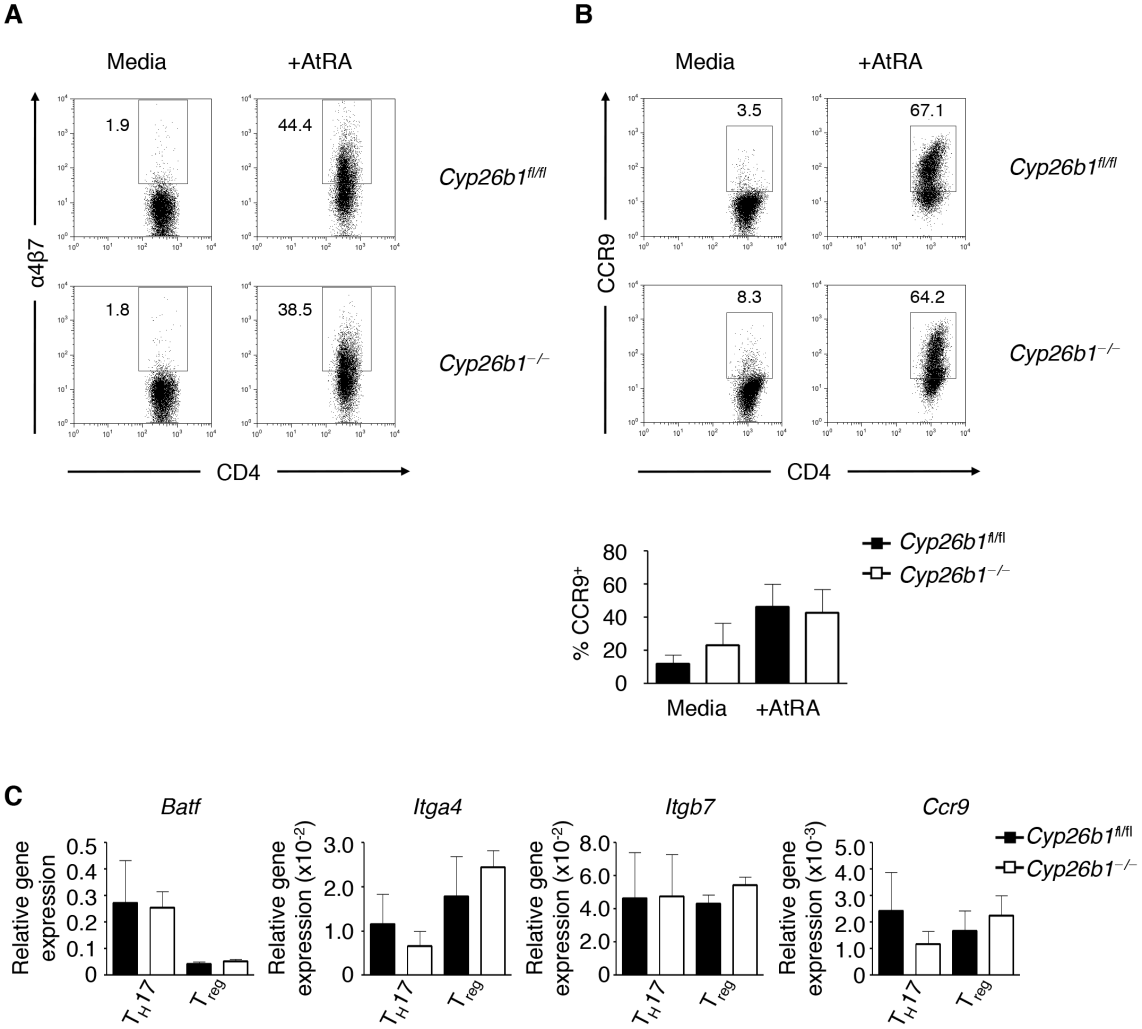
Deficiency in Cyp26b1 does not alter expression of intestinal homing molecules on T cells. (**A**) CD4^+^ T cells were isolated from *Cyp26b1* ^fl/fl^ and *Cyp26b1* ^−/−^ mice and stimulated with α-CD3/CD28 and IL-2 with or without 10 nM AtRA and α4β7 integrin expression was measured by flow cytometry. (**B**) CD4^+^ T cells were isolated from *Cyp26b1* ^fl/fl^ and *Cyp26b1* ^−/−^ mice were transiently stimulated with α-CD3/CD28 and IL-2 in the presence or absence of 10 nM AtRA to induce CCR9 expression measured by flow cytometry. (**C**) Gene expression of *Batf*, *Itga4*, *Itgb7*, and *Ccr9* was measured in isolated *Cyp26b1* ^fl/fl^ and *Cyp26b1* ^−/−^ T cells polarized under T_H_17- and T_reg_-promoting conditions (normalized relative to *Actb*). Data in (**A**) are from a single experiment; Data in (**B–C**) are representative of 3 independent experiments.

We next assessed whether *Cyp26b1^−/−^* T cells have an impaired ability to express intestinal homing molecules as a possible reason for why these T cells failed to cause disease in our colitis transfer model. Isolated *Cyp26b1* ^fl/fl^ and *Cyp26b1* ^−/−^ T cells showed no difference in integrin α4β7 surface expression after stimulation with or without AtRA **(** **Figure 5A** **)**. Isolated *Cyp26b1* ^fl/fl^ and *Cyp26b1* ^−/−^ T cells were also stimulated to express the RA-inducible chemokine receptor CCR9 but showed no differences in surface expression upon stimulation with AtRA **(** **Figure 5B** **)**. Further, *Cyp26b1* ^fl/fl^ and *Cyp26b1* ^−/−^ T cells polarized to T_H_17 and T_reg_ cell lineages showed no differences in gene expression of the homing molecules α4β7 and CCR9 or the transcription factor BATF recently been shown to regulate RA-induced expression of these homing molecules [18] **(** **Figure 5C** **)**. Thus, Cyp26b1 in T cells does not regulate RA-induced expression of intestinal homing molecules.

## Discussion

The Cyp26 family of enzymes are critical for limiting RA responses *in vivo*. For example, lack of Cyp26 during embryogenesis results in severe developmental defects [19], [20]. RA signaling in T cells has been shown to be regulated by Cyp26b1 [10]. Our study aimed to better characterize how control of RA metabolism and responsiveness by Cyp26b1 affects effector T cell differentiation and function *in vivo*. To do so, we generated a T cell-specific conditional knockout of *Cyp26b1* in mice. It is known that RA signaling is not required for normal hematopoiesis but can regulate precursors of the myeloid compartment [21]. On the other hand, deficiency in RAR signaling in T cells leads to significant activation defects [9]. However, an involvement of Cyp26b1-dependent RA metabolism during T cell development has not been investigated previously. It is known that infants exposed to retinoids *in utero* has been shown to develop malformations of various organs including the thymus [22]. We did not observe any gross developmental defects in *Cyp26b1* ^−/−^ mice, which were completely viable throughout adulthood. In terms of lymphoid development, *Cyp26b1* ^−/−^ mice displayed normal levels of CD4^+^ and CD8^+^ populations in the thymus, spleen and mesLN. Thus, our results suggest that RA metabolism in T cells has little effect on normal lymphoid development.

RA has paradoxical roles in controlling the balance between iT_reg_ and T_H_17 cell differentiation: RA promotes T_reg_ cell differentiation at the expense of T_H_17 cell development [16], [23], yet T_H_17 cells require physiological concentrations of RA for development and migration to the intestine [7]. Another study recently found that RA and TGF-β together induce histone modifications at the *Foxp3* locus that promote the stability of iT_reg_ cells [24]. Furthermore, the RA-inducible microRNA *miR-10a* was found to be expressed in both nT_reg_ and iT_reg_ cells, playing an important role in blocking the plasticity of T_reg_ cells [25]. Deficiency in Cyp26b1 led to increased frequencies of both iT_reg_ and T_H_17 cells suggesting that Cyp26b1 plays a role in limiting the differentiation of these T cell lineages. This effect was dependent on the presence of retinoids since serum-free culture conditions eliminated these differences, identifying a role for serum retinoids in T cell differentiation in the absence of Cyp26b1 *in vitro*. Based on our findings, we propose that Cyp26b1 can alter T cell sensitivity to endogenous retinoids during iT_reg_ and T_H_17 cell differentiation and plays a role in determining T cell responsiveness to RA.

We also identified a role for T cell-intrinsic expression of Cyp26b1 *in vivo*. We found that transfer of *Cyp26b1* ^−/−^ T cells into *Rag1* ^−/−^ mice led to a profound reduction in intestinal inflammation as compared to *Rag1* ^−/−^ mice transferred with *Cyp26b1* ^fl/fl^ T cells. Interestingly, we found no differences in the frequencies of iT_reg_ or T_H_17 cells in the mesLN and spleen. However, despite equivalent levels of *Cd4* gene expression in the intestine, we observed a reduction in the expression of *Il17a, Ifng* and *Tnfa* in the colon of *Rag1* ^−/−^ mice transferred with *Cyp26b1* ^−/−^ T cells, but no difference in levels of *Foxp3* gene expression. Thus, together with our observed comparable suppressive function between *Cyp26b1* ^fl/fl^ and *Cyp26b1* ^−/−^ T_reg_ cells *in vitro*, the role of Cyp26b1 in T_reg_ cell function in our T cell transfer colitis model is likely negligible.

The transcription factor BATF was recently shown to regulate the RA-inducible T_H_ cell expression of intestinal homing receptors [18]. To assess whether *Cyp26b1* ^−/−^ T cells have an impaired migratory ability, we characterized the ability of *Cyp26b1* ^−/−^ T cells to express RA-inducible intestinal homing molecules integrin α4β7 and the chemokine receptor CCR9 but found no altered capacity of surface expression. Similarly, expression of *BATF*, *Itga4, Itgb7,* and *Ccr9* were comparable in *Cyp26b1* ^fl/fl^ and *Cyp26b1* ^−/−^ T cells polarized to become T_H_17 and T_reg_ cells *in vitro*. Taken together, our results suggest that the role of RA metabolism in T cell function occurs predominantly in the intestinal tissues, consistent with the high levels of RA synthesizing enzymes expressed in the intestine [26]–[28]. Thus, the role of Cyp26b1 in promoting the development of T cell-mediated pathology in the intestine does not involve shifting the T_reg_/T_H_17 balance but potentially involves modulating effector T cell function at the site of inflammation.

In closing, we have shown that Cyp26b1 can limit the differentiation of iT_reg_ and T_H_17 cells and is differentially expressed by these lineages to fine tune RA responsiveness. Cyp26b1 in T cells was demonstrated to be required for the development of T cell-mediated chronic inflammation in the colon, potentially by regulating T cell effector function in the intestinal tissue. Thus, Cyp26b1 may serve as a novel therapeutic target to treat inflammatory bowel disease.

## Materials and Methods

### Ethics Statement

Experiments were approved by the University of British Columbia Animal Care Committee (Protocol number A11-0329) and were in accordance with the Canadian Guidelines for Animal Research.

### Mice

*Cyp26b1^fl/fl^* mice were generated asdescribed [13]. *Cyp26b1^fl/fl^* mice were crossed with *Cd4-*Cre mice to generate T cell-specific *Cyp26b1* ^−/−^ knockout mice. *Rag1* ^−/−^ mice were originally obtained from Jackson Labs. All mice were bred and maintained under specific pathogen-free conditions in house.

### T Cell Polarization and Flow Cytometry

Spleens and lymph nodes were passed through 70 µm strainers and pooled before isolation of CD4^+^ T cells using EasySep mouse CD4^+^ enrichment kits on a RoboSep (STEMCELL). 5×10^5^ cells were cultured onto plates coated with 1 µg/ml α-CD3/α-CD28 under either iT_reg_ cell- (10 ng/ml IL-2 and TGF-β), or T_H_17 cell- (1 ng/ml TGF-β, 10 ng/ml IL-1β, IL-6, IL-23, TNF-α, 10 µg/ml α-IL-4 and α-IFN-γ) polarizing conditions for 6 days; CTCM was the primary cell culture medium whereas X-VIVO 20 (Lonza) was used for serum-free cultures. Cells were stimulated with PMA (50 ng/ml), ionomycin (750 ng/ml), and brefeldin A (10 µg/ml) and stained for flow cytometry using the Foxp3/intracellular staining kit and fixable viability dyes (eBioscience); antibodies: CD4 (GK1.5), CD25 (PC61.5), CCR9 (eBio-CW1.2), Foxp3 (FJK-16s), α4β7 (FIB504), and IL-17a (eBio1787) (eBioscience). Samples analyzed using a LSR-II (BD Biosciences) and FlowJo software (Tree Star).

### T_reg_ Suppression Assay

CD4^+^ T cells were isolated from spleens and lymph nodes and a PE-selection kit (STEMCELL) was used to further isolate CD25^+^ T_reg_ cells. CD25^−^ conventional T cells (T_c_) were labeled with CFSE and plated in CTCM at 7×10^4^ cells/well in the presence of 10^4^ mouse T-activator beads (Gibco) and titrated with 2-fold increments of T_reg_ cells (T_c_:T_reg_ from 32∶1 to 1∶1). Percent suppression of T_c_ cells was measured by flow cytometry and was normalized against unsuppressed controls.

### RNA Isolation and Quantitative Real-time PCR

RNA was isolated from tissues by mechanical disruption and the TRIzol method (Ambion). RNA was purified from CD4^+^ T cells using RNeasy mini kits (Qiagen). Reverse transcription was used to generate cDNA and qPCR was performed using SYBR green primer sets. Reactions were run on an ABI 7900 real-time PCR machine (Applied Biosystems). Samples were normalized relative to *Actb*.

### T Cell Transfer Colitis

Spleens and lymph nodes from donor mice were processed for CD4^+^ T cell isolation. 4.5×10^5^ FACS-sorted CD4^+^CD45RB^high^CD25^−^ T cells were transferred into recipient *Rag1* ^−/−^ mice intraperitoneally to induce colitis. Weight loss and disease progression were monitored each week. Weight loss of 20% was considered the humane endpoint. Proximal colons were processed for histological hematoxylin and eosin staining and scored for disease (inflammatory cell infiltration, loss of epithelial architecture, thickening of colonic wall). Spleens and mesenteric lymph node (mesLN) cells were stimulated with 1 µg/ml α-CD3/α-CD28 overnight, then stimulated with PMA, ionomycin, and brefeldin-A for 5 hours, and were intracellularly stained for flow cytometry.

### Analysis of CCR9 Expression

CCR9 expression was induced as previously described using transient TCR and IL-2 stimulation [10]. 5×10^5^ isolated CD4+ T cells were stimulated with or without 10 nM all-trans retinoic acid (atRA) for 2 days (plates coated with 5 µg/ml α-CD3 and 1 µg/ml α-CD28) and then supplemented with 10 ng/ml IL-2 for an additional 2 days with antibodies removed. CCR9 surface expression was measured by flow cytometry.

### Statistics

Results are presented as mean ± SEM. Statistical significance between two groups was determined by unpaired Student’s *t*-test while comparisons between 3 or more groups were made by ANOVA with a Bonferroni post-hoc test using Prism software (GraphPad). Results were considered significant with a *P* value of <0.05.
